# The Effect of Altered Soil Moisture on Hybridization Rate in a Crop-Wild System (*Raphanus spp*.)

**DOI:** 10.1371/journal.pone.0166802

**Published:** 2016-12-09

**Authors:** Lesley G. Campbell, Kruti Shukla, Michelle E. Sneck, Colleen Chaplin, Kristin L. Mercer

**Affiliations:** 1 Department of Chemistry and Biology, Ryerson University, Toronto, Ontario, Canada; 2 Department of Biosciences, Rice University, Houston, Texas, United States of America; 3 Department of Horticulture and Crop Science, The Ohio State University, Columbus, Ohio, United States of America; Università Politecnica delle Marche, ITALY

## Abstract

Since plant mating choices are flexible and responsive to the environment, rates of spontaneous hybridization may vary across ecological clines. Developing a robust and predictive framework for rates of plant gene flow requires assessing the role of environmental sensitivity on plant reproductive traits, relative abundance, and pollen vectors. Therefore, across a soil moisture gradient, we quantified pollinator movement, life-history trait variation, and unidirectional hybridization rates from crop (*Raphanus sativus*) to wild (*Raphanus raphanistrum*) radish populations. Both radish species were grown together in relatively dry (no rain), relatively wet (double rain), or control soil moisture conditions in Ohio, USA. We measured wild and crop radish life-history, phenology and pollinator visitation patterns. To quantify hybridization rates from crop-to-wild species, we used a simply inherited morphological marker to detect F_1_ hybrid progeny. Although crop-to-wild hybridization did not respond to watering treatments, the abundance of hybrid offspring was higher in fruits produced late in the period of phenological overlap, when both species had roughly equal numbers of open flowers. Therefore, the timing of fruit production and its relationship to flowering overlap may be more important to hybrid zone formation in *Raphanus* spp. than soil moisture or pollen vector movements.

## Introduction

As climate change transforms environments [1], it can profoundly affect biotic processes occurring within those environments, such as species coexistence, migration and evolutionary trajectories [2–4]. If mating processes are affected by environmental variation [5], this can have numerous and cascading effects on fecundity [6, 7], the organization of genetic diversity within versus among populations [8, 9], and, ultimately, population persistence [10]. For instance, plant populations with more genetic diversity may be able to more rapidly adapt in response to environmental change than those with less diversity. Of all plant traits, mating systems are perhaps the most influential in structuring genetic diversity within and among populations by both transmitting diversity across generations and determining rates of diversity loss [11]. Plant reproduction can be extremely labile. For instance, autogamous plants (those that self-fertilize) may possess relatively lower heterozygosity and allelic diversity, whereas allogamous plants (those that outcross) may have relatively higher heterozygosity and allelic diversity [11]. Thus, environmentally-induced changes in plant mating patterns may alter the organization of intra- and inter-specific genetic diversity and could further magnify or dampen biotic responses to anthropogenic climate change [12]. Therefore, to develop a robust and predictive framework for the science of mating systems and gene flow, it is critical to understand how plant mating systems respond to environmental variation.

Elevated atmospheric levels of CO_2_ has increased the earth’s surface temperature [1, 13]. These climatic changes can strongly alter the availability of soil moisture to plants [13–18]. Most notably, changing climactic conditions have shifted geographic distributions by altering demography [19]. Researchers have repeatedly documented altered phenology and investment in reproductive structures in response to changing moisture regimes via the processes of adaptive evolution and phenotypic plasticity (e.g., [20–22]). These responses may impact the likelihood of interspecific hybridization, contribute new genetic material to recipient populations, generate novel phenotypes [23, 24], and initiate different evolutionary trajectories [25, 26]. Thus, hybridization rates may be indirectly influenced by environmental variation, including key abiotic variables influenced by global climate change.

Many studies of gene flow have attempted to identify key factors that contribute to successful hybridization (e.g., [27–29]). Similar to this study, most manipulate an experimental variable and measure the response of hybridization rate. However, it is critical to also examine the relationship between hybridization rate and traits that directly contribute to plant reproductive success and the probability of hybridization. For example, physical proximity, flowering phenology, investment in reproduction, and shared pollinators may partially drive hybridization rate to influence, at a small scale, the proportion of hybrid offspring, and, at a larger scale, the distribution and abundance of hybrid zones [27–35]. For plants, soil moisture can determine the size and abundance of flowers, and shift floral phenology [36, 37]. Further, hybridization rates depend on relative parental investment in male and female function—traits which commonly respond to water availability [38, 39]. Moreover, the number and size of flowers produced can alter pollinator communities and/or pollinator movement, which can influence the potential to hybridize [40–42]. For example, pollinators visit flowers with large corolla diameter (e.g., *Collinsia parviflora* [43]) or large numbers of flowers (e.g., *Raphanus raphanistrum* [44]) more frequently than plants with small corollas or few flowers. When floral display size of one or both species is affected by soil moisture, hybridization rates may shift in intensity (i.e., possibly causing swamping). Our recent work has documented that *R*. *raphanistrum* corolla diameter and flower production decreases with decreasing soil moisture [45, 46]. Thus, shifts in hybridization rates may be a consequence of a series of plant responses to local environmental conditions. Yet, despite the potential evolutionary consequences of hybridization, we know little about how environmental variation directly impacts mating systems, and thereby gene flow.

In this study, we used plants from experimental populations of the weedy annual *R*. *raphanistrum* and its cultivated relative (*R*. *sativus*) to investigate the effects of environmental variation (i.e., in soil moisture) on hybridization rates, as well as how these rates may be affected by local ecological context (i.e., neighbouring plants and insects responding to the same environmental variation). *R*. *raphanistrum* is a well-established model system in studies of plant evolution and ecology that has been used to evaluate the ecological consequences of crop-to-wild gene flow (e.g., [28, 47, 48]). However, few have investigated whether environmental variation affects interspecific gene flow rates, especially among crops and their wild relatives (but see [35, 49]). We estimated the response of hybridization rate between wild and cultivated radish to moisture, as well as other plant traits using a manipulative field experiment. We also explored how hybridization rate could be predicted by plant traits using a regression approach. Thus, we asked the following questions:

- What is the hybridization rate between crops and their wild relative, and under which environmental conditions is the hybridization rate maximized?
- Does soil moisture affect hybridization rate by altering plant size or age at reproduction, floral synchrony, or pollinator movement between parental species?

A basic understanding of how the environment, specifically soil moisture, is likely to impact hybridization rates is imperative to assess potential ecological consequences of climatic variation and global change. We discuss the potential implications of climate change for interspecific hybridization with a special focus on the introgression of crop alleles into weed populations.

## Methods

### Study Species

Cultivated radish (*R*. *sativus*) is an annual, cosmopolitan root vegetable. Wild radish, or jointed charlock (*R*. *raphanistrum* L.), is a closely related species, differentiated from the cultivar by a chromosomal translocation and a suite of morphological characters [50, 51]. Although *R*. *raphanistrum* flowers earlier than *R*. *sativus*, their flowering phenologies overlap [47] and they share pollinators [52]. Therefore, when the two co-occur, they can spontaneously hybridize, often producing localized hybrid swarms [53]. In rare instances, the hybrid swarms are persistent and become problematic weeds (e.g., [48, 50, 53]). Importantly, in the *Raphanus* crop-wild system, successful gene flow can be challenging and tends to occur in one direction only, with crop pollen fertilizing ovules on wild plants (i.e., crop-to-wild hybridization), for a number of reasons. First, fruits produced by crop plants remain attached to the maternal plant even after death, thus restricting seed dispersal of wild-to-crop hybrid plants to farm fields. Second, crop radishes are generally harvested before flowering making successful reproduction difficult for wild-to-crop hybrids. Nevertheless, crop plants are often allowed to flower before harvest in home gardens, which is the scenario we mimicked here.

As in Campbell et al. [54], we used flower colour, a simply inherited Mendelian trait, to distinguish hybrid from non-hybrid offspring. White flower colour stems from a dominant, crop-specific allele at this petal colour locus; yellow flower colour indicates a homozygous recessive genotype [50]. First-generation (F_1_) hybrid offspring are easily distinguished as they invariably produce white petals when derived from a *R*. *raphanistrum* mother with yellow petals. As plants flowered, we recorded their flower petal colour, which allowed us to estimate the proportion of plants with white flowers (hybrids) compared to yellow flowers (non-hybrids) in each bulk sample.

### Seed Source

The self-incompatible, *R*. *sativus* open-pollinated cultivar “Red Silk” (Harris-Moran Seed Co., Modesto, CA) acted as a cultivated pollen donor (homozygous for the dominant white flower petal colour allele). *R*. *raphanistrum* seeds, collected from a natural population close to Binghamton, New York, and grown in a common garden for several generations by the lab of Dr. J Conner in East Lansing, Michigan [55], acted as our maternal, weedy parent (homozygous for the recessive yellow flower petal colour allele). Given the self-incompatible mating system of *R*. *raphanistrum* and *R*. *sativus*, it was expected that these populations would be admixed and thus were representative of the genetic diversity occurring in both wild and crop populations.

### Experimental Design

In 2010, we established 36 experimental plots containing *R*. *sativus* and *R*. *raphanistrum* in agricultural fields on Waterman Experimental Farm at The Ohio State University in Columbus, OH, USA (*N*_sativus_ = 324, *N*_raphanistrum_ = 324). The field experiment was planned as a split-split plot design with nine blocks. Four watering treatments (further described below), including (1) Control Unsheltered, (2) Control Sheltered, (3) No Rain, and (4) Double Rain, were randomly applied to 2.4 x 3m plots within each of the nine blocks.

The experimental layout was conceived of as a split-split plot design. For this design, each moisture treatment was applied on the large plots; the two radish species were planted separately into two smaller subplots, each including nine individuals (i.e., either nine wild or nine cultivated radishes). Then each species had data collected on it three times; thus, date of collection was considered a sub-sub plot factor.

Experimental plots were spaced at least 200 m apart. We imposed four watering treatments that significantly altered soil moisture:

1. Control Unsheltered (CU), where rainfall was not manipulated;
2. Control Sheltered (CS), where, to understand the effect of the shelter on plants (separate from the effect of altered rainfall), rain-exclusion shelters funneled collected water into a 227L barrel. The collected rainwater was then applied to the plot within 48 hours of the rain event. Therefore, CS plots received the same amount of rain that fell in CU plots.
3. No Rain (NR), where rain-exclusion shelters intercepted all rainfall (although rain could and did blow in from the side of the structure) and the water was collected in a barrel;
4. Double Rain (DR), where rain-exclusion shelters intercepted the rain, which collected in a barrel; this rainwater was then applied to the plot within 48 hours of the rain event (identical to CS plots). In addition, the rainwater collected in a neighboring NR plot was also applied, which effectively doubled the soil moisture within the plot.

The rain-exclusion shelters were 1.52m in height on the gutter edge and 2.44m on the high edge of the roof. As reported in Campbell et al. [45], water manipulation treatments significantly and predictably altered the average soil volumetric moisture content (%VMC) within each experimental plot. No rain plots were approximately 50% drier than CS plots whereas DR plots were approximately 50% wetter than CS plots [45]. Finally, the %VMC of CU and CS plots did not differ significantly in 2010. Finally, there was a significant interaction between watering treatment and sampling date where %VMC of CS and CU plots tended to decline significantly over the summer, whereas NR plots tended to remain relatively dry and DR plots tended to remain relatively wet.

Seeds were planted on May 15, 2010, grown in a greenhouse for two weeks, and then seedlings were transplanted into tilled plots. We transplanted nine *R*. *sativus* on the south-western side and nine *R*. *raphanistrum* on the south-eastern side of each plot. Plants were spaced 46 cm apart. To ensure transplant survival, water was applied to all seedlings. After the first week, the experimental watering treatments were implemented for the remainder of the experiment. When assessed, we measured soil moisture three times at the center of each plot, using a TDR (Field Scout, TDR 100/200 Spectrum Technologies, Inc., Plainfield, IL, USA) at 10 cm depths. Measurements were made after each redistribution of rainfall, i.e., eight times during the growing season (between July 26, 2010 and September 28, 2010), and the plot-level estimate was averaged after these eight measurements. Weeds were removed when detected to ensure low levels of inter-specific competition and mimic garden plot conditions. However, this action may have also reduced inter-specific competition for floral insect visitors within the plot area (potentially increasing gene flow) or altered the attractiveness of plots to floral insect visitors (potentially reducing gene flow).

On the first day a plant flowered, we recorded plant age and stem diameter (an index of overall plant size). For each plot, the average days to first flowering and average stem diameter at first flowering was calculated for each species. On a weekly basis, we counted the number of open flowers on each plant within a plot. Across all weeks, the total number of flowers produced by all *R*. *raphanistrum* or all *R*. *sativus* plants within a plot was summed. The likelihood of hybridization is known to shift with the numerical advantage of plant ovules and pollen [56]. To estimate the relative availability of *R*. *raphanistrum* versus *R*. *sativus* hermaphroditic flowers, we calculated a weekly and overall index of relative flowering intensity (RFI) where the number of *R*. *raphanistrum* flowers was the numerator and the number of *R*. *sativus* flowers was the denominator. When RFI is greater than one, then *R*. *raphanistrum* flowers outnumber *R*. *sativus* flowers, and vice versa.

### Seed collection to estimate hybridization rate

To measure hybridization rate, we waited until plants of both species were flowering in the majority of plots to begin collecting seeds and then collected seeds on three dates (the Timing factor in analyses described below). To identify when, throughout the reproductive season, hybridization was taking place we allowed the plants to flower in synchrony for two weeks and then tagged (with a coloured plastic zip tie) two incipient fruits per flowering *R*. *raphanistrum* plant on July15^th^, 2012 (Early-season fruits). Then, we waited an additional two weeks, and tagged two additional incipient fruits per flowering *R*. *raphanistrum* plant on July 29^th^, 2010 (Mid-season fruits). Finally, after another two-week period, we tagged two additional incipient fruits per flowering *R*. *raphanistrum* plant on August12^th^, 2010 (Late-season fruits). All tagged fruits, along with two additional fruits produced directly above and/or directly below the tagged fruits (to ensure a similar date of flower pollination), were collected as they ripened and before dehiscence. Therefore, for each plant, we collected a total of six fruits per week, for a maximum of 18 possible fruits over the course of the season. We then removed the seeds from their siliques and stored bulked seeds from fruit collected simultaneously.

### Insect visitation

To assess the potential movement of *R*. *sativus* pollen to *R*. *raphanistrum* stigmas (i.e., movements that could have resulted in crop-wild hybridization), we monitored the movement of putative pollinators. Insect visits were monitored in each plot for 15 minute intervals between 8:00 and 16:00 h on spaced through the flowering season. We observed insect visitation activity for 88 of these 15 min intervals for a total of 22 h (n_CO_ = 17, n_CS_ = 24, n_NR_ = 27, n_DR_ = 20). During that time, 4423 insect visits were recorded.

At the beginning and end of each observation interval, we recorded the number and type of flying insects in the plot (i.e., excluding ants and spiders) and followed the movement of haphazardly chosen flying insects. A potential pollination “visit” was recorded each time an insect landed on a flower. The visited plant species and individual experimental plant identity were also noted. This allowed us to assess the potential for pollen movement between- versus within-species where we assumed that movement from *R*. *sativus* to *R*. *raphanistrum* by insect visitors were potential hybridization events that could be measured in *R*. *raphanistrum* progeny. From a potential pollination event, the type of insect was noted [57]. All insect visits were recorded during observational periods because visitation frequency was low. When an insect left the plot before the end of a given observational time period, we found another haphazardly chosen winged insect to monitor for movement until the 15-minute observation interval was over. From these data, we estimated the frequency of switching from *R*. *sativus* to *R*. *raphanistrum* (and not *vice versa*) by visiting flying insects, remembering that weedy plants in the *Raphanus* spp. complex are only generated when crop pollen fertilizes wild ovules. To explore whether pollinator movement responded to experimental watering treatments, we calculated the frequency of insect movement that could result in the production of hybrids on *R*. *raphanistrum* (i.e., from *R*. *sativus* to *R*. *raphanistrum*) relative to other possible movements (i.e., *R*. *sativus* to *R*. *sativus*, *R*. *raphanistrum* to *R*. *raphanistrum*, or *R*. *raphanistrum* to *R*. *sativus*).

### Common Garden to Assess Hybridization Rate

Seeds produced in 2010 were moved from Ohio to Ontario when the Campbell Lab relocated to Ryerson University, Toronto, Ontario. In 2011, we measured the rate of hybridization from crop-to-wild radish using 2010 *R*. *raphanistrum* progeny in a common garden at Koffler Scientific Reserve (KSR) in King City, ON, Canada (Hill (44°01’N, 79°32’W, 285 masl). In late May, up to ten seeds from each sample collected in 2010 were planted in 20 mL of PRO-MIX BX peat within plastic seedling trays and placed in a greenhouse at KSR (The number of total genotyped plants is available in S1 Table). After developing their first true leaves, seedlings were transplanted directly into the ground at 30cm within a cleared and tilled garden area at KSR. Plants were watered to ensure transplantation success after which no additional watering treatments (as in 2010) were applied. Plants from each 2010 plot were randomly distributed within the garden and were randomly transplanted, in a grid-like fashion. Assays for flower colour as an indication of hybridization rate were not negatively affected by location bias because flower petal colour is insensitive to environmental conditions [50, 54]. To protect plants from insect herbivory, plants were sprayed with the insecticide Malathion (Loveland Products Canada, Inc., Dorchester, ON, Canada) on June 21, 2010. Aphids were present at low densities later in the season but did not colonize any plant heavily. We successfully genotyped 8110 plants while 756 plants died before being genotyped. For each 2010 plot, the frequency of hybrids produced on *R*. *raphanistrum* plants was calculated, as both the frequency of hybridization events (0/1) and number of hybrid offspring relative to the total number of offspring.

### Statistical Analysis

We used PROC GLIMMIX in SAS Enterprise Guide (version 6.1) to employ generalized linear mixed models to predict the effect of watering treatments, species, time, and their interactions (as appropriate). For response variables we used frequency of hybridization events, frequency of hybrid offspring, days to flowering, stem diameter, overall abundance of flowers, relative flowering intensity (RFI), and pollinator movement. Our treatment factors were treated as fixed, while block and any interactions with block were deemed random. We used the option in Proc GLIMMIX to specify the distribution and corresponding LINK function of the data to improve model fit by choosing a distribution where the residuals best fit a normal distribution. As mentioned earlier, ours was a split-split plot design with watering treatment treated as a main plot factor, radish species as a subplot factor, and timing of fruit collection (i.e., early, mid or late) or the timing of moisture measurements as a sub-sub plot factor. However, analysis of some variables (e.g., hybridization rate or RFI) combined data for both species, so the timing factor were treated as a subplot factor (Table 1). Similarly, pollinator movement was simply tested for the effect of the watering treatment. Given the nature of the split-split plot design where treatments are applied at different scales, we used different errors to test our three factors and interactions therewith. We tested the effects of our main plot factor—watering treatment—using its interaction with block. Subplot factors (usually species, sometimes timing) and their interaction with watering treatment were tested with an interaction term including block, watering treatment, and the factor being tested. Split-split plot factors and interactions therewith were tested with the full four-way interaction between block, watering treatment, species, and collection time when degrees of freedom were available. In cases when we were limited in the number of degrees of freedom, we removed all factor interactions with block from the model.

**Table 1:** Results from generalized linear mixed effects models of the frequency of hybridization (0/1), abundance of hybrid offspring, life-history, phenology and pollinator visitation patterns in wild and crop radish (Species) grown under four watering treatments (Watering Treatment), with phenological overlap (Timing) sampled across the growing season (Date) performed using SAS PROC GLIMMIX.

*Response and parameter*	*df*	*F*	*P*
*Hybridization events* (Binary distribution, logit link function, n = 99)			
Watering Treatment	3,22	0.73	0.55
Timing	2,57	1.49	0.23
Watering Treatment x Timing	6,57	0.55	0.77
*Number of hybrid offspring* (Lognormal distribution, identity link function, n = 47)			
Watering Treatment	3,12	0.42	0.74
**Timing**	**2,12**	**4.59**	**0.03**
Watering Treatment x Timing	6,12	0.37	0.89
*Days to flowering* (Poisson distribution, log link function, n = 67)			
Watering Treatment	3,24	0.95	0.43
**Species**	**1,26**	**202.08**	**<0.0001**
Watering Treatment x Species	3,26	1.14	0.35
*Stem diameter* (Gaussian distribution, Identity link function, n = 68)			
Watering Treatment	3,24	0.58	0.64
**Species**	**1,27**	**204.94**	**<0.0001**
Species x Watering Treatment	3,27	0.33	0.80
*Overall abundance of flowers* (lognormal distribution, n = 546)			
Watering Treatment	3,24	0.17	0.9135
**Species**	**1,31**	**19.73**	**0.0001**
Watering Treatment x Species	3,31	0.21	0.8893
**Date**	**10,400**	**15.33**	**<0.0001**
Date x Watering Treatment	30,400	1.03	0.4240
**Species x Date**	**9,400**	**6.38**	**<0.0001**
Species x Date x Watering Treatment	26,400	0.77	0.7842
*Relative flowering intensity* (Gaussian distribution, Identity link function, n = 105)			
Watering Treatment	3,23	0.23	0.88
**Timing**	**2,62**	**9.86**	**0.0002**
Watering Treatment x Timing	6,62	1.24	0.30
*Pollinator movement* (lognormal distribution, Identity link function, n = 19)			
Watering Treatment	3,7	3.08	0.10

For the pollinator movement analysis, we lacked degrees of freedom to test the watering treatment x block interaction. For each response, underlying distribution, link function and sample size (n) are given. Significant effects are noted in bold.

Next, we tested the degree to which the frequency of hybrid offspring produced relative to the total number of offspring produced by *R*. *raphanistrum* was related to soil moisture, relative flowering intensity and/or the relative frequency of insect movement between parental species. To do so, we used PROC GLIMMIX to fit a multiple regression model. Here we expected that plots with higher frequencies of switching by insects, under wetter conditions, and with lower RFI would exhibit the highest hybridization rates and thus produce the most hybrid offspring.

## Results

### What is the Hybridization Rate between Crops and their Wild Relative?

**Fig 1:**
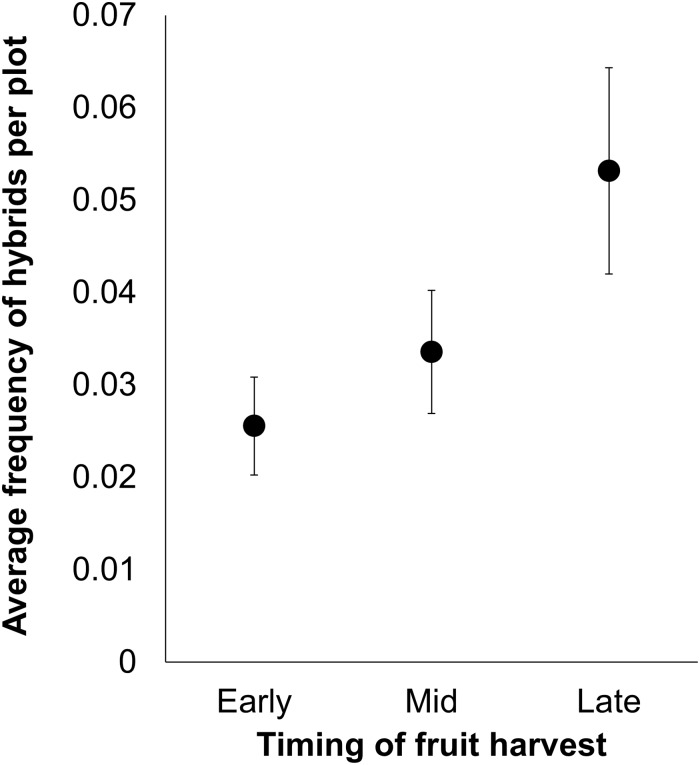
Least squares means (± 95% CI) of the frequency of *Raphanus raphanistrum* x *R*. *sativus* hybrid progeny produced as predicted by the date of fruit harvest (early-, mid- or late-season). Overall means for each treatment are represented by black bars.

Overall, hybridization was rare across all plots; of the 8110 progeny genotyped, only 114 total hybrids were produced (3.4% hybridization rate) by 55 maternal plants (of 279 surviving maternal plants assessed, which corresponded to 86% of originally planted *R*. *raphanistrum* seedlings). When hybridization occurred, the frequency of hybrid offspring produced could be predicted by the date on which seeds were produced (Table 1, Fig 1). There were significantly more hybrid offspring produced late in the season rather than early in the season.

### Does Soil Moisture Affect Hybridization Rate by Altering Plant Size or Age at Reproduction, Floral Synchrony or Pollinator Movement between Parental Species?

**Fig 2:**
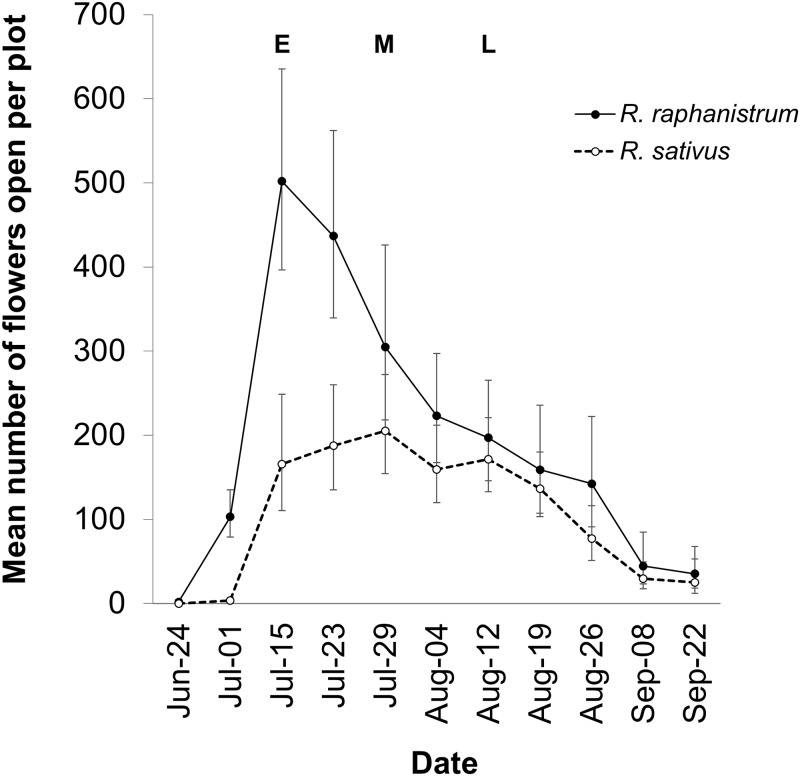
Least squares mean number of open flowers per harvest date (± 95% CI) across the flowering season of wild and crop radish, *Raphanus raphanistrum* and *R*. *sativus*, respectively. Early (E), Middle (M), and Late (L) sampling events are noted with arrows on the graph where developing fruits were tagged.

**Fig 3:**
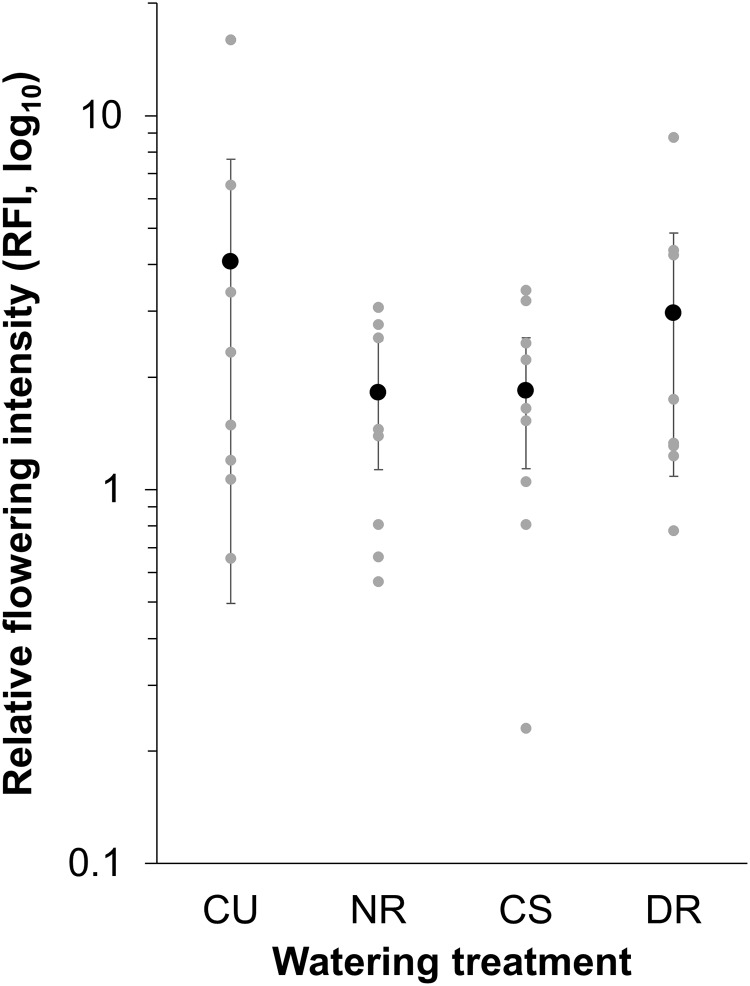
The effect of watering treatment (NR = No Rain, CU = Control Unsheltered, CS = Control Sheltered, DR = Double Rain) on relative flowering intensity (average number of open flowers per plot across a maximum of 12 sampling dates) of *Raphanus raphanistrum* compared to *R*. *sativus* across nine experimental blocks (grey dots). Values above 1 indicate more *R*. *raphanistrum* flowers relative to *R*. *sativus* flowers, whereas values below 1 indicate more *R*. *sativus* flowers relative to *R*. *raphanistrum* flowers. Least squares mean values (±95% CI) across plots within a watering treatment are represented by black dots.

As expected, *R*. *sativus* plants started flowering later and at a larger size than *R*. *raphanistrum* (Fig 2, Table 1). As a result, wild radish plants produced an average of 45% more flowers than crop radish plants, though the precise difference in floral abundance changed with census date (Fig 2). The greatest difference was found on the second sampling date between July 15 and July 29, 2010 where wild radish had 22–67% more flowers. Watering treatment did not significantly affect the date of first flowering, stem diameter at first flowering, overall abundance of flowers, or the relative flowering intensity of parental populations. Early in the season, there were more *R*. *raphanistrum* flowers than *R*. *sativus* flowers (as measured by RFI) whereas by the end of the season, the number of open *R*. *sativus* and *R*. *raphanistrum* flowers were roughly equivalent (Table 1, Fig 3).

Across the season, we observed an average of 1 movement per 15 minute observation bout where insects switched from *R*. *raphanistrum* flowers to *R*. *sativus* flowers (i.e., movements that could have resulted in crop-wild hybridization). The frequency of movements that could result in pollen movement from *R*. *raphanistrum* to *R*. *sativus* plants did not differ significantly across watering treatments (Table 1, S1 Fig).

We expected positive relationships between hybridization and pollinator movement and soil moisture, and a negative relationship between hybridization and RFI. The frequency of hybrid offspring in the total seeds produced by *R*. *raphanistrum* increased as RFI decreased (i.e., as *R*. *sativus* flowers became relatively more abundant, *β* = -0.041 ± 0.016, *n* = 34, *F*_1,22_ = 6.75, *P* = 0.016), but was not significantly related to insect movement (*P* = 0.55) or local soil moisture (*P* = 0.53, Table 2). Thus, RFI had the strongest influence on hybridization rate in this system.

**Table 2:** Models predicting hybridization rate between *Raphanus raphanistrum* and *R*. *sativus* based on plot-level soil moisture, relative flowering intensity, and the relative frequency that insect visitors switched from foraging on *R*. *raphanistrum* and went to *R*. *sativus* as predictors (for clarity the intercept (α) and error (ε) are included in all models).

Model	Δ*AIC*_*C*_	*w*	*Adjusted R*^*2*^	*β*_*Insect*_	*β*_*RFI*_	*β*_*H20*_
α+ln(RFI) +ε	0	0.54	0.13	-	**-0.397**	-
α +ln(RFI)+Water+ε	2.30	0.17	0.11	-	**-0.372**	-0.100
α+ ln(RFI) +Insect+ ε	2.58	0.15	0.11	-0.042	**-0.403**	-
α+Water+ε	4.68	0.05	0.01	-	-	-0.194
α+Insect+ln(RFI)+Water+ε	5.06	0.04	0.08	-0.028	**-0.377**	-0.096
α+Insect+ε	5.87	0.03	0	0.015	-	-
α+Insect +Water+ε	7.26	0.01	0	0.036	-	-0.198

When standardized regression coefficients (*β*) were significantly different from 0, the value is bolded.

## Discussion

Despite their sexual compatibility, these results suggest that inter-specific hybridization rates between crop and wild radish are spatially variable and sensitive to the relative abundance of open flowers of each parental species (RFI). For instance, the production of hybrid offspring occurred *more frequently* late in the reproductive season, or when the flowering intensity of crop and wild radish was approximately equal (equal numbers of open flowers for both species). Conversely, the production of hybrid offspring was *less frequent* when *R*. *raphanistrum* flowers were far more abundant than *R*. *sativus*, which occurred earlier in the reproductive season.

Contrary to our hypothesis, traits expected to be environmentally sensitive and thereby influence hybridization rates (e.g., plant size, RFI, overall floral abundance) did not respond to experimental manipulation of soil moisture. These results suggest that the timing of fruit harvest and its relationship to phenological overlap between parental species may be a stronger force in the formation of hybrid zones for *Raphanus* spp. than soil moisture or pollen vector movements. While this study suggests that species-specific temporal variation in relative flowering overlap predicts hybrid formation, it remains unclear how or if changes in the abiotic environment influence gene flow from crop-to-wild plant populations. Therefore, the consequences of anthropogenic climate change on evolutionary processes that maintain biological diversity, such as hybridization, remain unclear [58].

Spatially variable and difficult-to-predict rates of interspecific hybridization is a particularly important observation, since hybridization can occur within a single generation and has important evolutionary consequences [59, 60]. Several studies have documented changes in hybridization rates in response to natural environmental gradients in a diversity of organisms [34, 61–66]. Yet, to our knowledge, this is the first published description of a controlled, experimental manipulation of abiotic environmental variation on a population that subsequently explored the consequences for hybridization rates. This work reveals that hybridization rates can be difficult to predict based on manipulated abiotic conditions alone. However, once hybrids are formed, several studies have shown that their relative fitness is altered by variation in climatic conditions [35, 54, 67]. Experimental results presented in Campbell and Wendlandt [35] suggest that environmental conditions can play a significant role in realized hybridization rates in some species.

### Biotic Predictors of Hybridization

Hybridization rates typically respond to demographic differences in parental populations. On one hand, hybridization rates may be symmetric when parental populations are equally abundant. On the other hand, hybridization may be asymmetric when parental populations differ in abundance (e.g., [68–70]). Asymmetric hybridization events are thought to result from the relative abundance of male and female gametes from each parental population [56]. However, the evidence for the sensitivity of within-species hybridization rates to flowering effort is often incomplete and the causes are rarely experimentally attributed to differences in floral abundance between parental species. Here, we have documented that already low rates of hybridization (when parental populations produce similar numbers of flowers) decline further when one parental population produces far more flowers than the other. Despite many studies of hybridization between demographically asymmetric parental taxa (e.g., [68, 71, 72]), we are not aware of a study that has investigated the influence of relative floral abundance on patterns of hybridization. Floral production rates are known to vary through time and this variation appears to differ between crop and wild radish (and potentially between other crop-wild species complexes). Our results suggest predicting hybridization events may be more difficult across large geographic regions than previously recognized but may be most strongly influenced by the relative flowering intensity of both parental populations.

Pollinators often discriminate among flowers within and between species based on floral morphology, which can be highly sensitive to both abiotic and biotic variation (*R*. *sativus* [73]; *R*. *raphanistrum* [52]). Since both parental radish species are self-incompatible, only foraging events where pollinators move between plants result in seed production (especially since radish species were spatially separated in sub-plots, making it unlikely that wind or contact pollination could have occurred). Similarly, hybridization can occur only when foraging pollinators move between species. Furthermore, in an earlier study we have documented that increased water availability increased corolla diameter and potentially floral attractiveness [46]. We hypothesized that pollinator movements would correlate with actual hybridization events and thus rates of hybridization. Perhaps visiting insects did not respond to the microclimates within plots and thus did not influence gene flow.

### Introgression of Crop Genes into *R*. *raphanistrum*

Many factors could influence rates of hybridization between cultivated radish (*R*. *sativus*) and its wild, weedy relative (*R*. *raphanistrum*), but generally, risk assessments have only considered physical and phenological separation, as well as genetic compatibilities [28, 47, 74]. For instance, hybridization rates between cultivated and wild *R*. *sativus* have been shown to range from 14–100% and generally decreased with increasing distance, exhibiting a typical leptokurtic distribution [28]. Surprisingly, despite adjacent planting locations, our crop and wild plants exhibited strikingly lower rates of hybridization than previously reported [28]. However, long-term studies monitoring the frequency of crop alleles in advanced-generation hybrid populations would be necessary to determine whether crop alleles could persist in wild populations over longer periods of time, even in the face of challenging environmental conditions (e.g., [74]). Variation in environmental conditions could create opportunities for periodic introgression from crop plants and maintain polymorphisms within weedy populations.

## Supporting Information

S1 Table The number of F_1_ progeny genotyped based on flower colour for each plot and the number of mothers that contributed progeny to be genotyped per plot (maximum 9) during at least one collection point (Early, Mid or Late). The maximum number of progeny that could have been sampled per plot was: 540 seeds per plot. Progeny may not have been genotyped if the maternal plant did not flower on the sampling date, too few seeds per sample were produced, or the seed did not germinate or survive to flower before frost.
(DOCX)

S1 Fig The frequency of insect movements from crop to wild radish (*Raphanus sativus* and *R*. *raphanistrum*, respectively) across watering treatments (NR = No Rain, CU = Control Unsheltered, CS = Control Sheltered, DR = Double Rain) for nine plots per treatment (grey dots). Least squares mean values (±SD) across plots within a watering treatment are represented by black dots.
(DOCX)
