# Supplementary material for: The Effect of Altered Soil Moisture on Hybridization Rate in a Crop-Wild System (Raphanus spp.)
Source: PLoS One. 2016 Dec 9;11(12):e0166802. doi: 10.1371/journal.pone.0166802 (PMC5147839; doi:10.1371/journal.pone.0166802)
Supplement: S1 Table — The maximum number of progeny that could have been sampled per plot was: 540 seeds per plot. Progeny may not have been genotyped if the maternal plant did not flower on the sampling date, too few seeds per sample were produced, or the seed did not germinate or survive to flower before frost. (DOCX) [file pone.0166802.s001.docx]

**S1 Table.** The number of F_1_ progeny genotyped based on flower colour for each plot and the number of mothers that contributed progeny to be genotyped per plot (maximum 9) during at least one collection point (Early, Mid or Late). The maximum number of progeny that could have been sampled per plot was: 540 seeds per plot. Progeny may not have been genotyped if the maternal plant did not flower on the sampling date, too few seeds per sample were produced, or the seed did not germinate or survive to flower before frost.

| **Treatment** | **Plot** | **Number of progeny genotyped** | **Number of mothers contributing progeny** |
| --- | --- | --- | --- |
| Control Unsheltered | 1 | 330 | 9 |
|  | 10 | 210 | 9 |
|  | 13 | 252 | 9 |
|  | 20 | 349 | 9 |
|  | 23 | 311 | 9 |
|  | 25 | 152 | 9 |
|  | 32 | 211 | 8 |
|  | 35 | 259 | 9 |
| No Rain | 4 | 200 | 9 |
|  | 6 | 185 | 9 |
|  | 9 | 273 | 9 |
|  | 15 | 196 | 9 |
|  | 18 | 142 | 5 |
|  | 24 | 149 | 9 |
|  | 27 | 107 | 8 |
|  | 30 | 75 | 7 |
|  | 34 | 155 | 9 |
| Control Sheltered | 3 | 277 | 9 |
|  | 7 | 215 | 9 |
|  | 11 | 184 | 9 |
|  | 14 | 259 | 9 |
|  | 19 | 201 | 7 |
|  | 22 | 281 | 9 |
|  | 28 | 249 | 9 |
|  | 31 | 174 | 9 |
|  | 33 | 151 | 8 |
| Double Rain | 2 | 289 | 7 |
|  | 5 | 313 | 9 |
|  | 8* | 0 | 0 |
|  | 12 | 342 | 8 |
|  | 17 | 261 | 9 |
|  | 21 | 152 | 9 |
|  | 26 | 81 | 7 |
|  | 29 | 133 | 9 |
|  | 39 | 150 | 9 |

* Note: this plot did not have any synchronously flowering crop and wild plants.
