# Supplementary material for: The Effect of Altered Soil Moisture on Hybridization Rate in a Crop-Wild System (Raphanus spp.)
Source: PLoS One. 2016 Dec 9;11(12):e0166802. doi: 10.1371/journal.pone.0166802 (PMC5147839; doi:10.1371/journal.pone.0166802)
Supplement: S1 Fig — Least squares mean values (±SD) across plots within a watering treatment are represented by black dots. (DOCX) [file pone.0166802.s002.docx]

**S1 Fig.** The frequency of insect movements from crop to wild radish (*Raphanus sativus* and *R. raphanistrum*, respectively) across watering treatments (NR= No Rain, CU= Control Unsheltered, CS= Control Sheltered, DR= Double Rain) for nine plots per treatment (grey dots). Least squares mean values (±SD) across plots within a watering treatment are represented by black dots.
